# Using diffusion of innovation theory to understand the factors impacting patient acceptance and use of consumer e-health innovations: a case study in a primary care clinic

**DOI:** 10.1186/s12913-015-0726-2

**Published:** 2015-02-21

**Authors:** Xiaojun Zhang, Ping Yu, Jun Yan, Ir Ton A M Spil

**Affiliations:** School of Information Systems and Technology, University of Wollongong, Wollongong, 2522 Australia; Department of Industrial Engineering and Business Information System, University of Twente, Enschede, The Netherlands

**Keywords:** Consumer e-health, Internet, E-appointment scheduling, Online appointment service, Patient access, Diffusion of Innovation Theory

## Abstract

**Background:**

Consumer e-Health is a potential solution to the problems of accessibility, quality and costs of delivering public healthcare services to patients. Although consumer e-Health has proliferated in recent years, it remains unclear if patients are willing and able to accept and use this new and rapidly developing technology. Therefore, the aim of this research is to study the factors influencing patients’ acceptance and usage of consumer e-health innovations.

**Methods:**

A simple but typical consumer e-health innovation – an e-appointment scheduling service – was developed and implemented in a primary health care clinic in a regional town in Australia. A longitudinal case study was undertaken for 29 months after system implementation. The major factors influencing patients’ acceptance and use of the e-appointment service were examined through the theoretical lens of Rogers’ innovation diffusion theory. Data were collected from the computer log records of 25,616 patients who visited the medical centre in the entire study period, and from in-depth interviews with 125 patients.

**Results:**

The study results show that the overall adoption rate of the e-appointment service increased slowly from 1.5% at 3 months after implementation, to 4% at 29 months, which means only the ‘innovators’ had used this new service. The majority of patients did not adopt this innovation. The factors contributing to the low the adoption rate were: (1) insufficient communication about the e-appointment service to the patients, (2) lack of value of the e-appointment service for the majority of patients who could easily make phone call-based appointment, and limitation of the functionality of the e-appointment service, (3) incompatibility of the new service with the patients’ preference for oral communication with receptionists, and (4) the limitation of the characteristics of the patients, including their low level of Internet literacy, lack of access to a computer or the Internet at home, and a lack of experience with online health services. All of which are closely associated with the low socio-economic status of the study population.

**Conclusion:**

The findings point to a need for health care providers to consider and address the identified factors before implementing more complicated consumer e-health innovations.

## Background

Healthcare providers in Australia are currently facing a number of challenges, including the increasing size of the aging population, a shortage of healthcare workers, patient demands for increased access to health information and participation in healthcare decision making, and rising healthcare costs [1].

As a response to these challenges, there is a trend for healthcare organizations to provide consumer e-health services which allow patients electronic access to their medical information [2-4]. Consumer e-health has emerged with the rapid development of interactive consumer health informatics (CHI) and the increasing prevalence of the Internet [2]. It is described as **“**the use of modern computer technology and telecommunications to support consumers in obtaining information, analyzing their unique health care needs and helping them make decisions about their own health**”** and the **“**study, development, and implementation of computer and telecommunications and interfaces designed to be used by health consumers**”** [2,5]. Examples of consumer e-health include personal health records, smart cards, online health services, or engaging consumers in shared decision-making processes [2,6,7]. Currently, a substantial amount of e-health initiatives are in either the development or implementation phase [8,9], such as the Patient-Centered Access to Secure Systems Online (PCASSO) in the United States [8], or “Personally Controlled Electronic Health Record” (PCEHR), which is being implemented by the National E-Health transaction Authority (NETHA) in Australia [9]. According to the Australia National E-Health strategy, over the next 10 years, the electronic communication of health information will cover 90% of consumers or their care providers, and over 50% of them will be able to actively access and use electronic health records to manage their health and interact with health systems [10].

Although consumer e-health has the potential to facilitate patients’ access to healthcare services, there still remain some questions about whether patients are willing and able to accept and use them. A number of factors have been suggested as the determinants to predict patient acceptance of or resistance to consumer e-health services, including socio-demographic variables, device usability, awareness of the e-health innovations, and the user’s computer skills [11-19]. A systematic review of studies on patient acceptance of consumer-centered health information technologies (CHIT) reveals that major variables (67 of the 94 variables) associated with consumers’ acceptance of CHIT were patient factors [16]. These include socio-demographic factors, education level, prior experience of using computers, and health- and treatment- related variables [16]. In addition, human-technology interaction, prior experience of using computer/health information technologies and environmental factors appear to be significantly associated with patient acceptance of CHIT [16].

A meta-analysis by Dohan and Tan [17] of 15 articles recognizes that perceived usefulness is positively associated with a consumer’s intention to use web-based tools for health related purposes [17]. Another study on the impact of low literacy on the use of the internet for searching health information noted that, persons with low literacy made more mistakes during web-based searches and exhibited greater reluctance to access online health services [15].

Physical limitations for older adults to use e-Health services were also studied [18,19]. Choi reported that in the US, the rate of use the Internet for health related purposes by old adults is ranging from 32.2% in the 65–74 years old to 14.5% in the 75–84 years old [18].

According to Karahanna et al. [20], adoption and continued use of an IT innovation represent different behavioral intention [20]. IT adoption is the initial usage (new behavior) of an IT innovation at the individual level, whereas IT usage is the subsequent continued usage of an IT innovation after adoption at the individual level [20]. Consequently, factors determining user acceptance of an IT innovation differ from those affecting users’ attitudes toward continued usage of the IT innovation [20]. Therefore, it is important to distinguish these two concepts and investigate factors impacting on each of them.

Although many studies relating to patient acceptance of e-Health services have been conducted, to date, no attempt has been made to interpret and synthesize the evidence about factors influencing patient acceptance and use of consumer e-health applications in a primary health care context. In addition, there are significant concerns with a mismatch between what is supplied and what is demanded, which might hinder patient acceptance and use of e-health services, and lead to a loss of return on investment for healthcare organizations [21,22]. To that end, studies that examine the factors impacting on patient acceptance and use of consumer e-health applications are needed.

To bridge this knowledge gap, the current study focuses on investigating the factors influencing patients’ acceptance or ongoing use or dis-continuation of use of an exemplar consumer e-health service – a patient e-appointment scheduling service – through a longitudinal case study in a primary health care clinic. This study was a continuation of previous qualitative interview study [23]. A new data set extracted from computer log records adds a longitudinal view to this study. To increase the scientific value and generalizability, Rogers’ innovation diffusion theory was used as a theoretical lens to analyze the impact of factors on the patient attitudes toward the acceptance or rejection of the e-appointment service.

### E-appointment scheduling service as an IT innovation

One of the primary health care processes that is affected by increasing numbers of patients is the appointment scheduling process [24-26]. The traditional telephone-based appointment scheduling service is a time- and resource- consuming process – staff spend too much time on answering phone calls and managing appointments, which is inefficient [25,27]. In addition, telephone-based appointment scheduling requires patients to call the medical clinics during office hours, which can be inconvenient for patients who work full-time [27]. Therefore, it often results in congestion on the telephone lines and restricts the efficiency of the care providers’ work [26,27].

Another problem faced by clinics is that patients sometimes do not show up for their appointments. Missed appointments represent close to 10% of all appointments and this can lead to lower productivity for healthcare professionals and increased overall waiting-time for patients, which can decrease patient satisfaction and increase their health risks [28].

As a response to this challenge, more recently, some primary health care clinics have started to provide patients with e-appointment scheduling (EAS) services that enable a patient to conveniently and securely make appointments with healthcare providers through the Internet [27]. According to the classification of Antonia et al. [29], the EAS is a typical consumer e-health application: the use of the Internet for online health services.

In the healthcare context, patients can access EAS service through a web portal 24 hours a day and 7 days a week [27]. Once a patient’s preferred date and time are selected, the system will automatically confirm the patient’s appointment request and record the information in the database instantly without the involvement of care providers. In comparison with telephone-based appointment services, EAS enables patients to easily schedule their appointments. At the same time, by using this online scheduling tool, medical staff can identify new patients, allocate an appropriate time slot for each patient and easily manage patients’ appointments. Recently, Horvath et al. [30] reported a reduction of 2% in missed appointments for patients using an e-appointment system over two years [30]

With the prevalence of EAS in the health care sector, studies on patient acceptance and usage of EAS services have been conducted [31,32]. Cao et al. [31] conducted a qualitative study to examine patient usage of a web-based appointment system implemented in a Chinese public tertiary hospital [31]. Their study found that although many patients were not aware of the existence of the online appointment system, the use of the Internet for appointment making could significantly reduce the total waiting-time and improve patients’ satisfactions with outpatient services [31]. In addition, being ignorant of online registration, not trusting the Internet, and lacking the ability to use a computer were three main reasons given for not using the online appointment system [31]. Zhang et al. [23] also reported that, despite the benefits of using the e-appointment service, most patients in a tertiary hospital in Shanghai still registered via the traditional method of queuing, suggesting that health service providers should use a more effective method to promote and encourage patients to use the online system and improve their satisfaction with this service [32].

It is expected that through the study of the adoption and usage of this system, we can improve understanding of patient behavior in adopting and using consumer e-health applications and the factors that either influence acceptance or usage behavior.

### Theoretical basis

Rogers’ Innovation Diffusion Theory is one of the most popular theories for studying adoption of information technologies (IT) and understanding how IT innovations spread within and between communities [33,34]. According to this theory, innovation is an idea, process, or a technology that is perceived as new or unfamiliar to individuals within a particular area or social system. Diffusion is the process by which the information about the innovation flows from one person to another over time within the social system.

There are four main determinants of success of an IT innovation: communication channels, the attributes of the innovation, the characteristics of the adopters, and the social system [34]. The communication channels refer to the medium through which people obtain the information about the innovation and perceive its usefulness. It involves both mass media and interpersonal communication.

The attributes of an innovation include five user-perceived qualities: relative advantage, compatibility, complexity, trialability and observability [34]. Relative advantage is the degree to which the user perceives benefits or improvements upon the existing technology by adopting an innovation [34]. Compatibility captures the extent to which an innovation is consistent with the existing technical and social environment [34]. The more an innovation can integrate or coexist with existing values, past experience and the needs of potential adopters, the greater its prospects for diffusion and adoption [35,36]. Complexity measures the degree to which an innovation is perceived to be difficult to understand, implemented or used [34]. An innovation that is less complex is more likely to be rapidly accepted by end users [35,36]. Trialability is the ability of an innovation to be put on trial without total commitment and with minimal investment [34]. An innovation with higher trialability is more likely to be adopted by individuals [36]. Finally, observability is the extent to which the benefits of an innovation are visible to potential adopters [34]. Only when the results are perceived as beneficial, will an innovation be adopted [36].

Rogers has also characterized the individuals of a social system into five groups based on their attitudes toward an innovation: innovators, early adopters, earlier majority, later majority and laggards [34]. Innovators, representing 2.5% of the population in a social system, are the first group to adopt an innovation. According to Rogers, innovators have the ability to understand and apply complex technical knowledge essential for bringing in the innovation from outside the social system. The next group is the early adopters who are a more integrated part of the social system than the innovators. They tend to be well informed about the innovation, well connected with the new technologies and more economically successful [34]. The first two groups of adopters comprise 16% of the population in a social system. The next two groups, which account for 68% of the population of the social system, are earlier and later majority adopters. The last 16% of the individuals in the social system are called laggards [34]. They are the strongest resisters to the adoption of an innovation and most likely they tend to become non-adopters because of their limited resources and lack of awareness or knowledge of the innovation [34].

In Rogers’ theory (2003), a social system is “a set of interrelated units engaged in joint problem solving to accomplish a common goal” [34]. It constitutes a boundary within which the diffusion of innovations takes place [34]. Rogers suggests that the structure of a social system affects the individuals’ attitude toward the innovation, and consequently, the rate of adoption of innovations [34].

In recent years, diffusion of innovation theory has been used to study individuals’ adoption of new healthcare information technologies [37-43]. To name a few, Helitzer et al. applied the diffusion of innovation theory to assess and predict the adoption of a telehealth program in rural areas of New Mexico [37]. Chew et al. used innovation diffusion theory to study use of Internet healthcare services by family physicians [38]; and Lee conducted a qualitative study using Rogers’ theory to investigate the adoption of a computerized nursing care plan (CNCP) by nurses in Taiwan [39].

These studies demonstrated that Rogers’ innovation theory is useful for conceptualization of technology adoption in the context of e-heath. Therefore, this theory was used in the study as the theoretical framework to examine and explain the impact of factors, in particular, the characteristics of innovations and innovation decision-making processes, on patient acceptance and ongoing usage of an EAS service.

## Methods

### Research setting

The case study was conducted in a primary health care centre, Centre Health Complex (CHC), located in Shellharbour, a suburban town on the South Coast of New South Wales (NSW), 100 kilometers south of Sydney. The medical centre provides family medical practices, specialist medical services, allied health services and wellness services to the local community. The staff included 19 physicians (17 GPs and 2 nurse practitioners), 7 allied health professionals, 10 specialists and 7 clerical front office staff.

According to the Australia Bureau of Statistics (ABS) 2011 census data, 63,605 people resided in the town where the study was conducted [44]. Of these, 49% (N = 31,158) were male and 51% (N = 32,447) were female [44]. The average age of the population at the study site was 37 years [44]. People aged between 18 and 64 years made up 71.9% (N = 45762) of the population and people aged 65 years and over comprised 19.7% (N = 12576) of the population [44].

In addition, the ABS census data also suggested that 57.1% of the population at the study site reported working full-time, lower than the average of 60.2% in New South Wales (NSW) and 59.3% in Australia [44]. On the other hand, the unemployment rate was 13.2%, which was higher than the average level in NSW (11.6%) and the whole country (11.5%) [44]. The average weekly personal income of the study site was $479, lower than the average level of NSW ($561) and whole country ($577) [44]. Therefore, the study site had a relatively low socioeconomic status in NSW and in Australia.

### Design and implementation of the patient e-appointment scheduling service

In CHC, the current phone-call based appointment system was often congested and could not provide prompt services to patients. A patient e-appointment scheduling service was identified by the CEO of CHC as urgently needed in order to relieve the congestion of the phone-call based appointment system and provide patients with the opportunity for ‘self-service’ 24 hours a day and 7 days a week.

**Figure 1:**
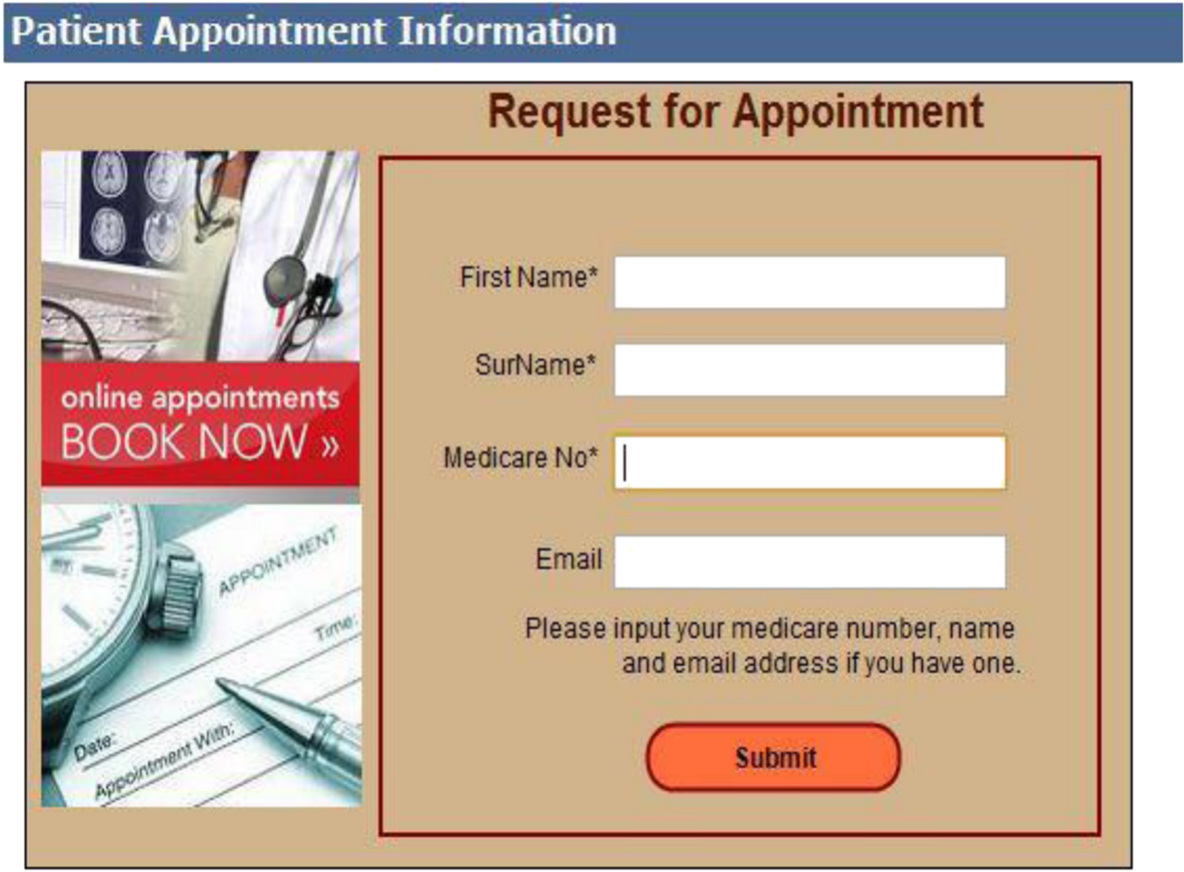
**Patient login web page.**

The e-appointment service was developed and installed on a server at CHC at the end of January 2011. A web link was placed on the home page of the medical centre and a click on it directed the user to the e-appointment service. Figure 1 shows the patient login web page.

**Figure 2:**
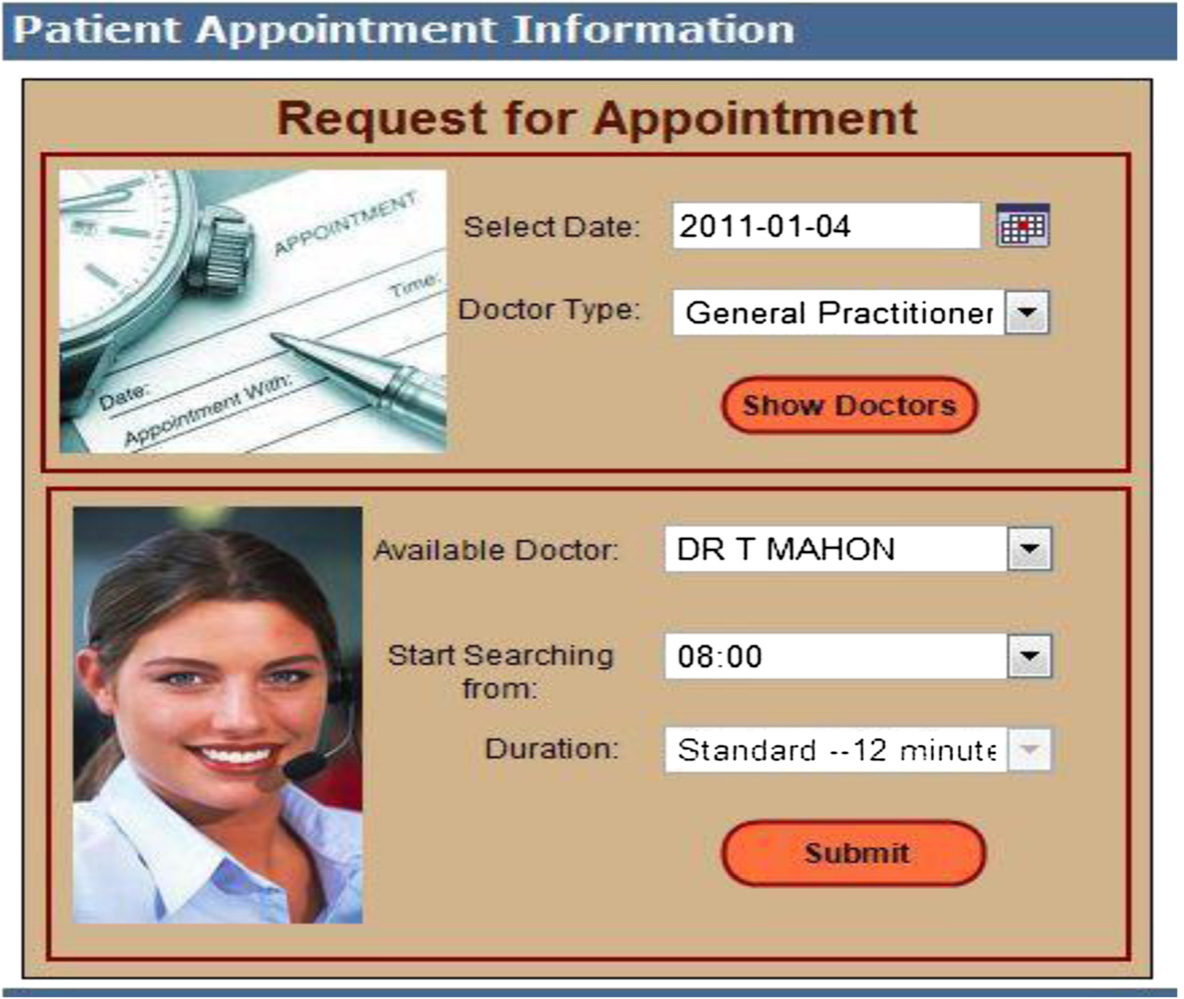
**Online appointment options web page.**

Once successfully logged in to the online appointment system, patients could select their preferred appointment date, time and doctors, as shown in Figure 2.

**Figure 3:**
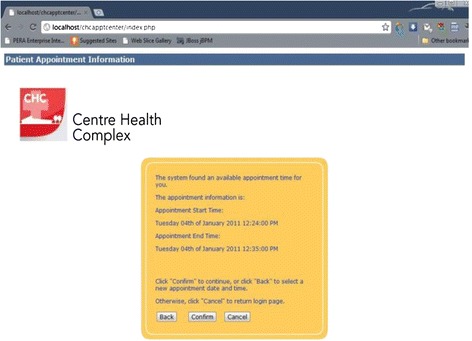
**Appointment confirmation web page.**

After patients made their choice, a confirmation web page with print function would be displayed. The confirmation web page provides patients with the opportunity to reconsider their choices before the information is finally sent to the server database. After a final choice was made, a confirmation e-mail was generated automatically and instantly sent to the e-mail address provided by the patient. This e-mail contained detailed appointment information, including the patient’s name, doctor’s name, appointment date, time and confirmation number. In comparison with a phone-call based service, the online appointment system had the advantage of allowing patients to instantly review and print out their appointment information. Figure 3 shows the appointment confirmation web page.

Information about the e-appointment service was disseminated to patients through the following channels: (1) fliers left at the reception desk; (2) posters placed in prominent locations in the medical centre; (3) an advertisement on the CHC web site, and (4) a voice message played during the phone call waiting periods was implemented 6 months after online system implementation. The information disseminated included the web link of the e-appointment service and the steps to follow to make an appointment using it.

At the time of the field study, the CHC provided a patient with three options for appointment making, include phone-call, online self-service and walk-in.

### Methods for data collection and analysis

#### Methods for data collection

This study used both qualitative and quantitative research methods. To obtain detailed, in-depth qualitative data, a semi-structured interview was conducted. Six major issues were captured in each interview: (1) the patient’s basic demographic information, including age, education level and employment status, (2) the variation of continued usage of the online appointment service over the whole study period, (3) their awareness of the e-appointment service and the communication channels through which the information was received, (4) their perceptions of the e-appointment service compared with phone-call based appointment making, (5) prior experience of using online healthcare services, and (6) their intention to use the e-appointment service in the near future.

This study was sponsored by the University Research Committee (URC) Internal Industry Linkage Grant Scheme. The survey was approved by the University of Wollongong/South Eastern Sydney & Illawarra area Health Service Human Research Ethics Committee. The semi-structured interview guide was reviewed by the owner of the medical centre, the practice manager and a general practitioner (GP). It was then trialed on three patients to ensure they understood all the questions and could provide relevant answers to these questions. Afterwards, the interviews were conducted in the medical centre from April 2011 to May 2013.

### Interview procedure

The first survey was conducted three months after the system was implemented, from April to June 2011. The time of the survey was decided based on the research group’s experience with other e-Health system implementation studies, which was also confirmed by Munyisia et al. [45]. In order to understand whether a patient’s perception of the system would change with time, the survey was repeated three times, from June to August 2011, from October to November 2012 and again from April to May 2013.

In each interview, the first author approached patients who were sitting in the waiting area, appearing not to be engaged in any activities. The researcher explained the purpose and procedure of the interview, then gave an information sheet with written explanation to the patient. Only after oral consent was given by the patient, would an interview start. Each interview lasted about 10 to 15 minutes and was audio-recorded with the interviewee’s permission. The interview stopped when theoretical saturation was reached [46].

For the protection of the patient privacy, each interviewee was given a unique number with the form of ‘PID_’, followed by three digital numbers. For example, ‘PID_001’ represents the first patient who participated in the interview.

### The procedure for computer log data collection

The computer log data provides a complete and accurate longitudinal data set about patients’ ongoing or dis-continued use of the e-appointment service. Therefore, in addition to the interview, appointment log data was collected from the online appointment database. The online appointment database was built based on Microsoft SQL Server 2008. It stores each patient’s online appointment information, including date, time and the name of the GP to be visited. A set of data searching/results export SQL programs were developed and used to extract the online appointment information from different data tables. The search results were automatically exported to the Microsoft Excel worksheet, which was further used for data analysis.

Computer log data collection was conducted from January 2011 to May 2013. Twenty nine months of appointment log records were captured and analysed to ascertain the patients’ usage of the EAS.

### Interview data analysis

Following the qualitative data analysis technique suggested by Miles and Humberman [46], each interview was transcribed from verbatim into a word processing document [46]. The transcribed data was then carefully read and divided into meaningful analytical units that were relevant to the research aims [47]. By using the method proposed by Zhang et al. [48], the analytical unit was identified and a code was assigned to signify this particular unit [47,48]. Each meaningful unit was coded into different sub-categories and then grouped into the categories that were framed based on Rogers’ innovation diffusion model. For example, for the question “which method do you prefer to use to make an appointment”, one interviewee responded that “I would prefer to use the phone because I prefer to speak to someone and confirm”. This statement was coded as “prefer phone-call for oral communication and confirmation”. Another interviewee answered “I will probably use the phone. I found it is easier to use the phone” was coded as “prefer phone-call because of its ease of use”. Both units were placed in the category of “preference for phone-call”, but with different sub-categories “prefer for oral communication” and “phone-call is easier than e-appointment service”. This process was applied repetitively to all of the transcribed data until the overall coding was completed [47,48].

Each interview was double-checked in order to prevent a patient from being repeatedly interviewed in different survey periods. Therefore, although the interview data was collected in four stages, the qualitative interview study was not treated as longitudinal study.

Statistical analysis was conducted in SPSS20 in order to assess the influence of demographic factors on perceptions. Spearman’s correlation test and a Chi-square test were conducted to measure associations and differences in proportions between groups. Statistical significance was set at P-value < 0.05.

### Computer log data analysis

In order to investigate patients’ continued usage of the EAS, qualitative thematic analysis with coding via Microsoft Excel was used to analysis the computer log data. The analysis results were categorized and coded based on Roger’s innovation-decision model and the topic guide. For example, one patient registered as an online appointment user but never used this service during the whole study period, this patient was coded as ‘logged into the web site but never used’. Where a patient used the electronic, as well as the phone-call/walk-in appointment service, more than once, this patient was coded as ‘used both online and phone-call services’. In total, the online appointment users were categorized into four groups, including (1) logged into the web site but never used, (2) tried once but never used again, (3) used both online and phone-call services, and (4) only used online appointment system.

## Results

### Demographics of the participants and their use of the e-appointment service

Fifty-one patients were interviewed in the first survey. In the three follow-up surveys, 20, 32 and 22 patients were interviewed, respectively. This gave a total number of 125 interviewees, providing sufficient variation in age, gender and social status of the study population.

Table 1 provides an overview of the demographic profiles of the interviewees and patients recorded in the appointment database. During the four periods of face-to-face survey, 125 patients between the ages of 18 to 78 years participated in the interview (see Table 1). These included 61 men (49% of the interviewees) and 64 women (51% of the interviewees). The average age of the interviewees was 38.7 years (SD 16.04 years). Accordingly, 75.2% of respondents (N = 94) were aged between 18 and 64 years, and 24.8% of respondents (N = 31) were aged 65 and above. A comparison of the participants’ demographic profile with the ABS census data suggests that the sample was representative of the population in the study site.

**Table 1:** **Basic demographic profiles of interviewees and patients recorded in appointment database**, **and their use of phone**-**call or online system to make appointment**

		**Usage of each type of appointment method by % (** **No.)** **of Interviewees**		**Usage of each type of appointment method by % (** **No.) ** **of Patients recorded in the database**	
		**Using phone-** **call/** **walk-** **in only**	**Using online appointment service**	**Using phone-** **call/** **walk-** **in only**	**Using online appointment service**
Age	18-29	86% (30)	14% (5)	91% (6402)	9% (631)
	30-41	81% (26)	19% (6)	91.5% (5211)	8.5% (485)
	42-53	89% (24)	11% (3)	95.5% (5003)	4.5% (234)
	54-65	100% (18)	−	96% (3842)	4% (160)
	Above 65	100% (13)	−	98.8% (3604)	1.2% (44)
Gender	Male	90% (55)	10% (6)	95.3% (11195)	4.7% (557)
	Female	87.5% (56)	12.5% (8)	92.8% (12867)	7.2% (997)
Education	Primary/Secondary/TAFE	92% (99)	8% (9)	−	−
	University	71% (12)	29% (5)	−	−
Work status	Full time	83% (54)	17% (11)	−	−
	Part time	100% (26)	−	−	−
	Unemployed	91% (31)	9% (3)	−	−
Total		89% (111)	11% (14)	94% (24062)	6% (1554)

Eleven percent of the interviewees (6 males and 8 females) used the e-appointment service in all four survey periods. This was much higher than the real number of online appointment users suggested by the computer log records stored in the database of the medical centre (see Table 1). There was no significant gender difference in terms of preferred method for appointment making by either interviewees or computer log data. Six interviewees who used the e-appointment service at least once were in the age group of 30 to 41 years, representing 19% of the population in this age group. Five interviewees were between18 to 29 years of age and 3 users between 42 to 53 years of age. None of the online appointment users was above 54 years of age.

According to the computer log records, from January 2011 to May 2013, 25,616 patients visited the medical centre through phone-call, walk-in or online appointment making services. Only 6% of them (N = 1554, 557 males and 997 females) had continuously used the e-appointment service to make appointments to see their doctors over the whole study period.

Of the interview participants, 29% of the 17 interviewees (N = 5) with a university degree used the e-appointment service at least once. Of the remaining 108 interviewees (86.4% of the total respondents) who reported having a primary, secondary or certified technical education degree from the Technical and Further Education (TAFE) system in Australia, only 8% (N = 9) used this online service at least once.

The relationship between educational level and online service usage was assessed by Spearman’s correlation analysis. The result suggests that usage of the EAS by male interviewees had a weak, yet significant positive correlation with their educational level (r_s_ (59) = 0.3, P = 0.031). However, no such correlation was found for the female interviewees (r_s_ (62) = 0.17, P = 0.064).

The interview data shows that 52% of the interviewees (N = 65) reported working full-time, which was found to be similar to the ABS census data presented above. 21% (N = 26) worked part-time, and the remaining 27% of interviewees (N = 34) were unemployed, which was found to be higher than that reported in the census data (13.2%).

The results also show that 17% of the interviewees (N = 11) who worked full-time had experience of using the e-appointment service. No part-time workers reported using the system. The other 3 online system users came from the unemployed group, accounting for 9% of this population. A strong, positive correlation between employment status and usage of e-appointment service was found for male interviewees (r_s_ (59) = 0.44, P = 0.012). However, no such association was found for female interviewees (r_s_ (62) = 0.12, P = 0.234).

### Variations in patients’ continuous usage of the EAS over two and a half years

**Figure 4:**
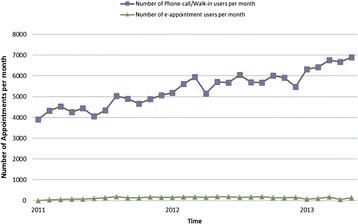
**Overall usage of phone**-**call**/**walk**-**in and online appointment services from January 2011 to May 2013.**

In order to examine if patients’ perceptions of the EAS changed over time, the computer log data that reflects the continued usage of two modes of appointment making: phone-call/walk-in versus e-appointment service, was collected and compared in the running chart across the entire study period (see Figure 4). The top line shows the monthly number of visiting patients who used phone-call/walk-in services to make appointments to see their doctors. It can be seen that the number of phone-call/walk-in patients per month had gradually increased from 3906 to 6897 patients over two and a half years, and the average number was 5367 patients per month (SD 832 and CI 95% = 5064–5670). The flat line at the bottom of the Figure shows the monthly number of patients who used the EAS at least once. The average number was 128 patients per month (SD 49 and CI 95% = 110–146). It can be seen that the number of patients using the online self-service remained unchanged, even slightly reduced in 2013. This is because the online system had been shut down several times for server maintenance.

**Figure 5:**
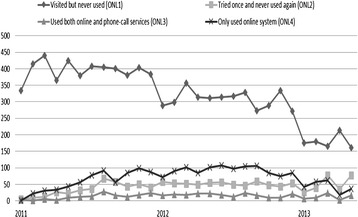
**Overall usage trend of the online appointment service by registered users over twenty**-**nine months of field study** (**from January 2011 to May 2013).**

In order to investigate patients’ usage patterns, online appointment users were further split into four categories (see Figure 5): (1) ONL1: logged into the medical centre web site but never used the online appointment service. On average, there were 321 patients per month (SD 80 and CI 95% = 292–350) in this group; (2) ONL2: used the online appointment system only once and continued making appointments by phone-call appointment thereafter. The average number was 44 patients per month (SD 18 and CI 95% = 38–50); (3) ONL3: used the online appointment system more than once, but also used the phone call-based system. The average number was 14 patients per month (SD 7 and CI 95% = 11–17); and (4) ONL4: always used the online appointment system. The average number was 69 patients per month (SD 29 and CI 95% = 59–79).

The detailed number of each type of users was given in Table 2. It can be seen that at the first data point (from January to December 2011), 5978 patients logged into the online appointment web site. Among these online users, more than 79% (N = 4737) persisted in phone-call/walk-in appointment making, and 6.8% (N = 407) used the online appointment service only once and never used it again. Among the remaining 14% of online users (N = 834), 18% (N = 147) used both the online service and phone-call/walk-in for appointment making, and 82% (N = 687) used the online system only for making an appointment.

**Table 2:** **Categories of online system users recorded in the computer log records at each data point** (**from January 2011 to May 2013**)

	**% (No.)** **registered patients at each data point in computer records**		
**Usages of online appointment system by registered users**	**January to December 2011%** **(N)**	**January to December 2012%** **(N)**	**January to May 2013%** **(N)**
1. Logged into web site but never used	79.2% (4737)	65% (3696)	63% (895)
2. Tried once and never used again	6.8% (407)	11% (624)	18% (256)
3. Used both online and phone-call services	2.5% (147)	4% (212)	4% (60)
4. Only used online system	11.5% (687)	20% (1110)	15% (217)
Total	100% (5978)	100% (5642)	100% (1428)

At the second data point (from January to December 2012), 5642 patients logged into the online appointment web site (see Table 2). In comparison with the first data point, the number of patients who preferred to use the online service (in category ONL3 and ONL4) had significantly increased to 1322, accounting for 23% of the total online users. The number of patients in each category remained similar at the third data point (from January to May 2013).

### Patient awareness of the EAS and effectiveness of communication channels for disseminating the information

In the first survey period, only 22% of the interviewees (N = 11) were aware of the existence of the EAS (see Table 3). The number increased substantially to 55% (N = 11) four months after system introduction. It increased to 59% (N = 19) one year later, and then dropped to 23% (N = 5) two years after the implementation of the EAS. It can be seen that there was an increasing trend of awareness of the EAS over the one and a half years of the survey period. Simultaneously, the percentage of online service users among interviewees increased from 5.8% to 20% from the first data point to the second and remained similar at the third data point. However, more than 60% of the interviewees remained unaware of the EAS over the entire survey period. Spearman’s rank-order correlation revealed that there was a strong, positive correlation between interviewees’ awareness and usage of the EAS (r_s_ (39) = 0.467, P < 0.001).

**Table 3:** **Percentage of interviewees who were aware of and used the e**-**appointment service at each data point**

**Survey period**	**% (No.)** **of interviewees**	
	**Aware of the online system/** **total Interviewees**	**Used the online service/** **total interviewees**
April 2011 – June 2011	22% (11/51)	5.8% (3/51)
July 2011– August 2011	55% (11/20)	20% (4/20)
October 2012–November 2012	59% (19/32)	19% (6/32)
April 2013 – May 2013	23% (5/22)	5% (1/22)
Total	37% (41/125)	11% (14/125)

Those interviewees who were aware of the EAS reported receiving the information about the availability of this service through visiting the medical centre web site or through the voice message heard when making an appointment via phone. No interviewees appeared to notice the posters or fliers placed at the locations that were assumed to be prominent in the medical centre.

### Interviewees’ perceptions of e-appointment service

#### Perceived advantages of the EAS

Twelve out of fourteen interviewees (86%) who used the EAS at least once stated that the service was easy to use. In comparison with the phone-call based system, the e-appointment service provided certain advantages such as after-hour access to the medical appointment service and less waiting time.

### Less waiting time

Eleven out of fourteen interviewees (79%) who used the e-appointment service at least once agreed that they could schedule an appointment as soon as they needed it. One patient said:

*“The online system gives your available time slots, or just straightway what’s available and what’s not.” [Patient 15]*

### Providing after-hour service

**Figure 6:**
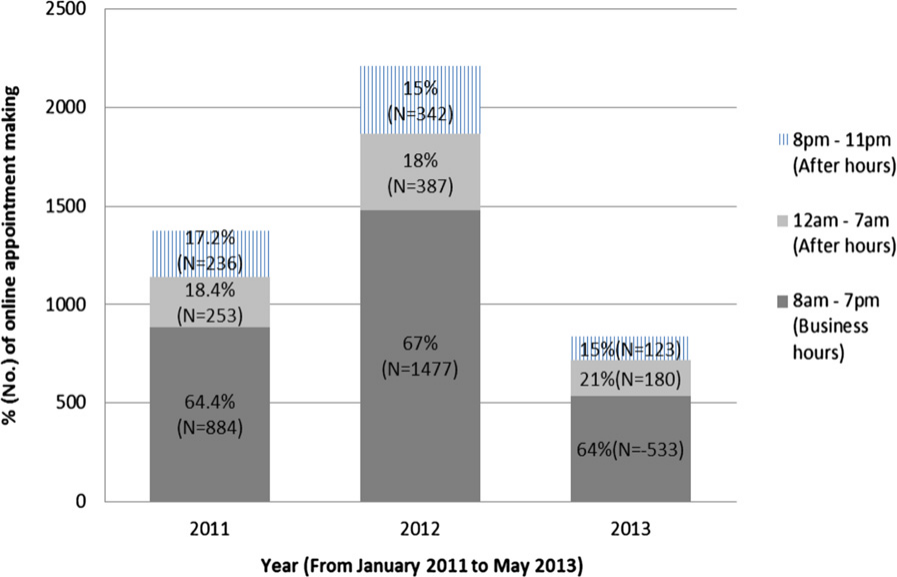
**Usage of the e**-**appointment service by patients during each period** **(2011 and 2012**: **January to December**, **2013**: **January to May).**

With the phone call-based appointment service, after-hour appointment requests were diverted to a message recorder in the medical centre, and the patient was advised to call back during office hours. The EAS provided patients with the opportunity for “self-service” available 24 hours a day and 7 days a week. From January 2011 to May 2013, 4415 appointments were made through the e-appointment service, 34.5% (N = 1521) of them were made after hours, and the remaining 65.5% (N = 2894) were made during the period 8 am to 7 pm, which were the business hours of the medical centre. Of those after-hours online appointment requests, 54% (N = 820) were lodged during the period 11 pm to 7 am, and another 46% (N = 701) during the period 8 pm to 11 pm. The percentage of online appointments made during business hours and after-hours are presented in Figure 6. It can be seen that, in each year, more than 60% of the online appointments were made during business hours, 18-21% were made during the period 12 am to 7 am, and 15-17% of online appointments were made between 8 pm and 11 pm.

### Perceived disadvantages of the EAS

The interview data suggests that inflexible time slot allocation and an insufficient number of appointment selection options were the main disadvantages of the EAS as perceived by the interviewees.

### Inflexible time slot allocation

Inflexible time slot allocation was reported to be the major disadvantage of the e-appointment service. Five out of fourteen interviewees (36%) who used the online service at least once recommended that the time slot allocation should be more specific. For example, one interviewee suggested that:

*“The appointment times are very limited. It seems there is only one appointment time for the online customer which is always 12 minutes past the hour. A few more choices would be helpful.” [Patient 43]*

Where a patient’s initial preference could not be met, the patient was required to choose a different date, time or doctor. Four interviewees suggested that the service should support “find doctors who meet desired time and date” or “display all available time slots for a specific doctor”, as one interviewee said:

*“Very good that you don’t have to ring up, but we should be able to see what doctors are available at the time you pick instead of having to go back if it’s not the right time for you.” [Patient 27]*

### Insufficient options provided by e-appointment service for appointment making

Four out of fourteen interviewees (29%) who used the e-appointment service at least once suggested that this service should provide management options for making online appointments. One interviewee said:

*“[The online service] doesn’t allow you to cancel the appointment. It would be helpful as I cannot always get to the phone easily.” [Patient 65]*

Similarly, another interviewee suggested that

*“[To add] the ability to manage your booking would be nice, just in case you need to cancel an appointment or change the time.” [Patient 19]*

### Patients’ prior experience of using online healthcare services

Among all interviewees, the youngest age group (18–29 years) used the Internet the most for health-related purposes (54%, N = 19). Use of the Internet for health-related purposes appeared to decrease with increasing age. Those aged above 65 years had the lowest rate of Internet usage (15%, N = 2), as shown in Table 4.

**Table 4:** **Percentage of interviewees who reported to have or not to have had prior experience with online healthcare services**

		**% (No.)** **of interviewees**	
		**Searched online health information**	**Using e**-**appointment service at least once**
Age	18-29	54% (19/35)	14% (5/35)
	30-41	50% (16/32)	19% (6/32)
	42-53	41% (11/27)	11% (3/27)
	54-65	33% (6/18)	−
	Above 65	15% (2/13)	−
Total		43% (54/125)	11% (14/125)

The interview data suggests that 76% (n = 41) of these online healthcare service users searched for general health-related information, such as information about the common flu, vaccinations, side effects of new medications and suggestions for healthy food. The remaining 24% (N = 13) had searched for diseases information of concern to them, such as information about kidney-stents, cancer, symptoms of heart disease or mental health problems.

Although 43% of interviewees had prior experience of using the Internet for health-related purposes, more than 50% of the interviewees reported their preference for obtaining information from their doctors rather than from searching the Internet. They believed doctors could provide more accurate and credible information than the Internet.

### Patients’ intention to use the e-appointment service in the near future

The percentage of interviewees who intended to use the EAS at each age group was given in Table 5. In total, 25.6% (N = 32) of respondents expressed their intention to use the online system next time to see a doctor. The remaining 74% of respondents (N = 93) preferred to use the phone-call based service. The reason why those patients persisted in making phone appointment was given in the previous study [23]. These include the perceived advantages of easy of use, preference for communication with and putting trust in a person, low computer literacy level or Internet skills, and lack of access to a computer or the Internet at home.

**Table 5:** **Percentage of interviewees who intended to use the e**-**appointment service**

		**Would like to use the online system in the near future** ** % (** **N)**	
		**Male**	**Female**
Age	18 - 29	31% (4)	42% (8)
	30 - 41	38% (5)	37% (7)
	42 – 53	23% (3)	16% (3)
	54 – 65	8% (1)	5% (1)
	Above 65	−	−
Total	100% (13)	100% (19)	

### Patients who did not have a computer or Internet access at home

About 30% of the interviewees (N = 28) who preferred to use the phone-call appointment service reported that they did not have a computer or Internet connection at home, therefore making an appointment by phone or walking-in was their only choice. Of these patients, 64.3% (N = 18) were unemployed and 21.4% (N = 6) worked full-time, respectively. The remaining 14.3% (N = 4) worked part-time. The relationship between work status and computer/Internet connection at home was examined by Chi-square test. A significant association was found between employment status and Internet access at home (P < 0.001). It implies that patients who were not in the labour force were less likely to have an Internet connection at home than those who worked full-time or part-time.

## Discussion

The computer log records show that the monthly adoption rate of the EAS increased slowly from 1.5% (76/4941 patients/month) at three-months after system implementation, to 4% (287/7189 patients/month) at twenty-nine months. The monthly number of patients using the EAS was steady, compared to the increasing number of patients who used phone-call/walk-in appointment services at the end of study period (see Figure 4). Computer log records also show that, although more than 300 patients visited online appointment web site each month, most of them were still not ready to accept this e-health innovation. In total, only 6% (1554/25,616) of patients continuously used the e-appointment service to see the doctors in the clinic during the whole period of the study. The overall adoption rate of the EAS was still lower than the ‘take-off’ point – 13% of the overall population according to Rogers’ innovation diffusion theory [34]. Therefore, at the end of the study, only the ‘innovators’ had adopted the online service.

As suggested by Rogers [34], the communication channels, the attributes of the e-appointment service, the characteristics of the patients who were the consumers of the online system, and the social system, had all contributed to the low adoption rate of the online service.

### Influence of communication channels on patient adoption of the e-appointment service

In this study, the information about the availability of the e-appointment service was carefully planned and disseminated to patients through mass media channels, include posters, fliers and web advertisement. However, twenty-nine months after system implementation, only 5% of appointments were made through the EAS. The majority of patients were not aware of the existence of the online service and consequently, could not use it for appointment making.

In response, a new communication channel – a voice message played during the phone call waiting periods was implemented 6 months after implementation. This appeared to be an effective channel that helped to increase patient awareness of the online appointment service to a certain extent, as suggested by the 55% of the interviewees who reported that they were aware of the existence of the online appointment system at the second data point (see Table 3). However, twenty-nine months after system implementation, despite the introduction of the voice message, only 23% of the interviewees reported being aware of the availability of the online system. It appears that the majority of patients did not pay attention to the voice-message, which can be seen as an example of mass media in Rogers’ terms [34].

It implies that the use of mass media was not effective in attracting patients’ attention to the availability of the e-appointment service. Obviously, lack of awareness of the existence, features and benefits of the e-appointment service had a negative impact on patient adoption of the new e-health system, as validated by the result of the correlation between the interviewees’ awareness and usage of the e-appointment service. This fact confirms the view of Cao et al. [31] that effective dissemination of information about any new online technology could improve the usage of the innovation. This lesson is useful to learn for other consumer e-health initiatives, so that more effective and personalized communication strategies can be developed and used to increase patient awareness of a new e-health service.

### Influence of the perceived attributes of the EAS on its adoption and use

According to Rogers (2003), there are four perceived attributes of the EAS which might influence patient adoption and use of the service. They are relative advantages, compatibility, complexity and trialability.

### Relative advantages

The interview results show that the extended after-hour service and less waiting time appeared to be the main attributes attracting patients to adopt the e-appointment service.

However, more than 88% (N = 111) of the interviewees expressed their preference for using the phone call appointment service (see Table 1). From their perspective, the e-appointment service was inferior to making an appointment by phone. This was because the phone-call service provided them with an immediate, fast and convenient way to access the appointment service compared with the EAS. Besides, the phone-call service provided an opportunity for patients to chat with a person – the receptionist, who could make a more flexible decision on the spot. They saw no personal advantages in making appointments online. Lee et al. also suggested that patients’ need for human interaction may hinder their adoption of online self-service [49].

Furthermore, the e-appointment service did not provide patients with any other value-adding services, such as access to patients’ electronic healthcare records. Therefore, there was little or no value for patients to switch to the e-appointment service.

### Compatibility

A major reason for patients to continue with making appointment by phone was its compatibility with their preference of having a conversation with a person, the opportunity to discuss the options for more complex situations and to receive reliable information from their doctors. Tradition and habit also appeared to play an important role in hindering adoption of the EAS. It is the first time that this factor has been reported in the studies on the adoption of consumer e-health innovations [50]. It might help to increase the probability of patient adoption of the EAS if the system can integrate a voice message similar to the receptionist talking to patients addressing their real-time concerns.

### Complexity

Although the e-appointment service was perceived as easy to use for those patients who had continued to use this service, a large number of patients in this study had never accessed the Internet at home. Some did not even have a computer or Internet access at home. Only a small portion had prior experience of using the Internet for health-related purposes. As a result, more than 74% of the interviewees (N = 93) did not feel confident about their ability to use the Internet or the e-appointment service.

### Trialability

In this study, the computer log records showed that 45% of the registered users stopped using the system after a trial use. There might be several explanations for this: (1) the patient did not need to see the doctor again after the appointment; or (2) they directly made the follow-up appointment after seeing a GP in the clinic and thus had no further needs to make appointments online; or (3) they preferred to make appointments by phone or in person rather than using the e-appointment service. Karahanna et al. [20] also indicate that trialability appears to be a less important factor in determining an individual’s decision to continuously use an IT innovation after individuals adopt the innovation [20].

In general, the interview results suggest that a high level of relative advantages and low level of complexity are the factors which will encourage patients to adopt this e-appointment innovation. However, the computer log data shows that the e-appointment service is still in the initial knowledge stage of the innovation-decision process, and only the ‘innovators’ in the patient population adopt and continuously used this innovation by the end of the field study.

### Influence of the patients’ characteristics on their adoption of the EAS

In this study, the patients’ social and demographic characteristics, including age, education level and work status, appeared to have influenced their choice of use or non-use of the e-appointment service. Computer log records showed that 72% of the ‘innovators’ (N = 1116) were in the age group of 18 to 41 years.

The reason why the patient group who worked full-time were more likely to use the e-appointment service might be that this group had difficulty making phone calls during office hours, and could only do so after-hours. In this case, the online service might be helpful. Thirty six percent of the online appointment users (N = 5) had a university degree, suggesting that that the younger patients with a higher educational level and better job prospects are more likely to adopt consumer e-health services than older, less educated patients with fewer job opportunities.

In addition to the social and demographics factors, as shown in Table 1, all of the patients who reported using the e-appointment service had prior experience using the Internet for health-related purposes. LaRose and Eastin found that users’ Internet self-efficacy is positively affected by their prior Internet experience, positive outcome, and Internet usage [51]. Macpherson et al. [19] also reported that lack of access to the computer or Internet, or low computer/Internet skills could have a negative impact on the acceptance and use of e-Health services by older adults [19]. Therefore, having prior experience of in using online health care services also appears to be positively associated with patient acceptance of the e-appointment service.

### Influence of the nature of the social system on the adoption of consumer e-health

According to the Australia Bureau of Statistics’ 2011 census data, the population of the study site had a lower income and higher un-employment profile in comparison with the national demographic data [44]. The interview results suggest that this population group is yet to develop the capacity and interest in using the Internet for health-related purposes. As explained by Rogers’ Innovation Diffusion Theory, the probability of patients adopting the e-appointment service was negatively influenced by their lower socio-economic profile.

### Limitations of the study

The research was conducted in a regional area in Australia, therefore the findings may only be comparable to a similar population group. Qualitative studies in other suburban areas would enrich the results of the study and provide a better understanding of patients’ adoption of consumer e-health innovations.

Another limitation in sampling is that the patients who were sitting in the waiting area and appeared to be willing to communicate with researchers were more likely to be invited to participate in the interview. This trend was revealed by a much higher adoption rate of 13% from interview results than the 6% of population consistently suggested by the computer log records. The bias in the interview results was effectively rectified by the computer log records. In addition, this study was conducted based on a particular form of consumer e-health application – the online appointment service. Therefore caution is needed in generalizing the relevance of the findings from this study to other types of consumer e-health applications in similar or other healthcare settings. Further substantial studies are needed to understand patient behavior in adopting more complicated consumer e-health innovations.

## Conclusion

This study found that adoption and usage of an e-appointment service in a primary care clinic was low after the service had been introduced for 29 months. Several factors appeared to have contributed to this low rate of adoption of the e-health innovation. These included ineffective communication of the availability of the e-appointment service to the patients, a perceived lack of value of the new online service for the majority of patients, the incompatibility of the new service with the patients’ preference for oral communication, and some functional limitations of the service itself. In addition, lack of access to a computer/the Internet at home, low computer literacy levels, and the low socio-economic status of the study population also appeared to be factors causing the low rate of adoption of the new online service. Conversely, the e-appointment service was perceived to be advantageous for those patients who worked full-time and could only make an appointment to see a doctor after business hours.

The findings of this study were in accordance with Rogers’ four determinants of success of innovations. Communication of the new e-health initiative to patients appears to be difficult. This challenge cannot be underestimated for any similar e-health initiatives.

This study provides valuable insight about the feasibility of introducing consumer e-health services in a primary health care setting. The findings point to a need for health care providers to consider and address the identified factors before the implementation of more complicated consumer e-health services, such as PCEHR in Australia. Further researches can be conducted on other types of consumer e-health services and the optimal implementation strategies that could lead to successful adoption, usage and benefits realization.
